# Assessment of Myocardial Ischemia Using Coronary Postmortem Computed Tomography Angiography Based on the Voronoi Algorithm: A Case Report

**DOI:** 10.7759/cureus.64565

**Published:** 2024-07-15

**Authors:** Haruki Fukuda, Hiroyuki Tokue, Miyuki Shiraishi, Akira Hayakawa, Rie Sano

**Affiliations:** 1 Department of Legal Medicine, Graduate School of Medicine, Gunma University, Maebashi, JPN; 2 Department of Diagnostic Radiology and Nuclear Medicine, Graduate School of Medicine, Gunma University, Maebashi, JPN; 3 Department of Forensic Sciences, Graduate School of Medicine, Akita University, Akita, JPN; 4 Department of Forensic Medicine, Faculty of Life Sciences, Kumamoto University, Kumamoto, JPN

**Keywords:** voronoi algorithm, postmortem ct angiography, myocardial infarction, autopsy, 3d reconstruction

## Abstract

Postmortem computed tomography angiography (PMCTA) is a valuable tool for diagnosing vascular conditions, such as hemorrhages, in trauma cases. This case report demonstrates the use of the Voronoi algorithm to assess myocardial ischemia using coronary PMCTA. A male in his 70s was found unconscious in a car after colliding with a traffic light pole. Despite medical interventions, including pericardial drainage and cardiopulmonary resuscitation, the patient died two hours later. PMCTA revealed significant filling defects in the left anterior descending artery (LAD), consistent with plaque rupture and narrowing observed during autopsy. The cause of death in this case was likely cardiac tamponade due to cardiac rupture secondary to myocardial infarction resulting from LAD stenosis. Cardiac perfusion areas were analyzed using the Voronoi algorithm, demonstrating a total myocardial volume of 151.9 mL in the left ventricle. Perfusion volumes were calculated as 92.9 mL (61.2%) for the LAD, 34.2 mL (22.5%) for the left circumflex artery, and 24.9 mL (16.4%) for the right coronary artery. The predicted ischemic volume distal to the LAD stenosis was estimated to be 49.8 mL (32.8%). Furthermore, the ischemic areas observed during autopsy macroscopically corresponded well with the predicted ischemic regions. This case highlights that combining PMCTA with the Voronoi algorithm provides an accurate method for assessing myocardial ischemic areas, offering a non-invasive approach to visualize and quantify perfusion and ischemic regions.

## Introduction

Postmortem computed tomography angiography (PMCTA) is widely recognized in forensic medicine for its effectiveness in diagnosing hemorrhages in trauma cases, owing to its detailed examination of the vascular system [1]. The PMCTA technique encompasses both whole-body angiography using a heart-lung machine [2,3] and selective methods, such as targeted coronary angiography [4-8]. Targeted coronary PMCTA can be performed using two injection methods: one involves injecting a contrast agent into the aorta before autopsy [6,9], while the other introduces the contrast agent directly into the coronary artery after the heart has been removed during autopsy [4,5,10]. Selective coronary PMCTA is particularly useful for assessing the degree of coronary artery stenosis and occlusion, and it can reveal characteristic changes associated with anomalies, such as anomalous aortic origins of coronary arteries and hypertrophic obstructive cardiomyopathy [4,10].

In clinical practice, coronary CTA reportedly assists in patient risk assessment and decision-making through myocardial segmentation based on the Voronoi algorithm [11]. The Voronoi method is a mathematical algorithm that divides a region or space based on the shortest distance to a specified set of points or lines [12]. By applying the Voronoi algorithm to coronary CTA data, it becomes feasible to calculate the perfusion volume of the myocardium per coronary artery and identify the extent and location of ischemic myocardium relative to the total myocardial volume. Ide et al. demonstrated a significant correlation between the myocardial volume quantified automatically using a Voronoi-based algorithm and actual perfusion volume measured in swine specimens [13]. However, there have been no reports linking predicted areas of ischemic myocardium derived from PMCTA data using the Voronoi algorithm to the specific locations of ischemic changes observed during autopsy. In this case study, we aimed to explore this correlation.

## Case presentation

A male in his 70s was found unconscious inside a car that had collided with a traffic light pole. Upon arrival at the hospital, he underwent pericardial drainage and cardiopulmonary resuscitation (CPR) but unfortunately died approximately two hours later. Approximately two liters of blood were collected during pericardial drainage. Subsequent investigations revealed that the decedent's vehicle had moved from a stationary position, traversed a red light, and collided with a pole at an estimated speed of 20 km/h. The patient had a history of type 2 diabetes.

PMCT performed 46 h after death revealed blood accumulation in the pericardial cavity, bilateral rib fractures, and a hematoma around the spleen. Subsequent autopsy, performed 1 h after the CT scan, recorded the decedent's measurements at 175 cm in height and 61.2 kg in weight. External examination revealed the treatment scars of the medical intervention, including CPR and pericardial drainage. No significant subcutaneous hemorrhage or injury was observed throughout the body. Internal examination revealed a 120 mL hematoma within the pericardial cavity. Fractures of the anterior walls of the left and right ribs were observed. Given the symmetry of the fractures and the minimal soft tissue hemorrhage, it was concluded that they were attributable to CPR rather than a collision with a traffic light pole. Additionally, approximately 80 mL of blood was found in the abdominal cavity, without any associated damage to the abdominal organs. Blood loss was considered a result of therapeutic interventions, including pericardial drainage.

**Figure 1:**
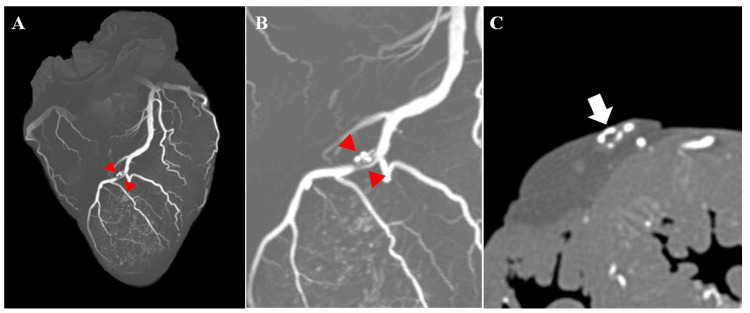
Coronary postmortem computed tomography angiography (PMCTA) images. (A) Maximum intensity projection (MIP) image in front view. A filling defect in the left anterior descending (LAD) artery is observed, as indicated by the red arrowheads. (B) Enlarged view of panel A. (C) Axial image of the LAD artery in the section corresponding to the red arrowheads in (A). A filling defect is evident, as indicated by the white arrows.

**Figure 2:**
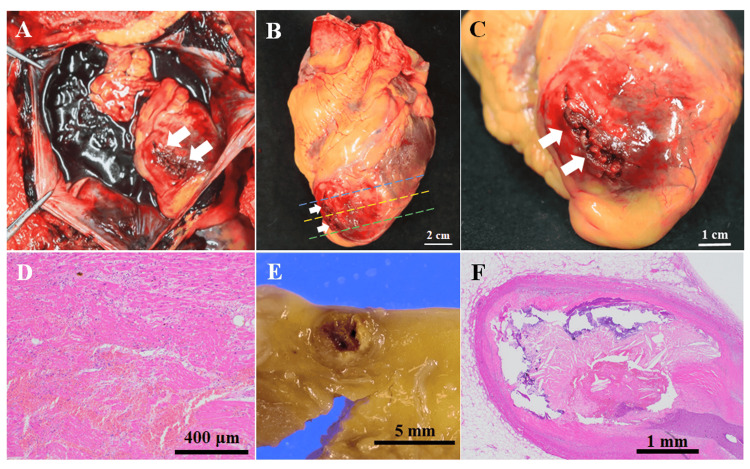
Macroscopic and microscopic observation of the heart. (A) Photograph of the pericardium during autopsy. There is a rupture in the left ventricle indicated by a white arrow, along with a hematoma in the pericardial cavity. (B) Image of the heart prior to formalin fixation. The left ventricular rupture, indicated by the white arrows, is observed. The blue dashed line indicates cross-sections shown in panel A of Figure 3. The yellow dashed line indicates the cross-section shown in panel D of Figure 3. The green dashed line represents the cross-section shown in panel G of Figure 3. (C) Enlarged view of the apex of the heart. Rupture of the left ventricle is evident, indicated by the white arrows. (D) Microscopic image of the area surrounding the rupture (hematoxylin and eosin staining, ×4). Hemorrhage, coagulative necrosis, and proliferation of fibroblasts are observed. (E) Cross-section of the left anterior descending artery (LAD) artery. Narrowing is observed, consistent with the occlusion observed in the maximum intensity projection (MIP) image. (F) Histopathological image of the LAD artery corresponding to panel C (hematoxylin and eosin staining, ×2). Plaque rupture and narrowing with calcification are observed.

**Figure 3:**
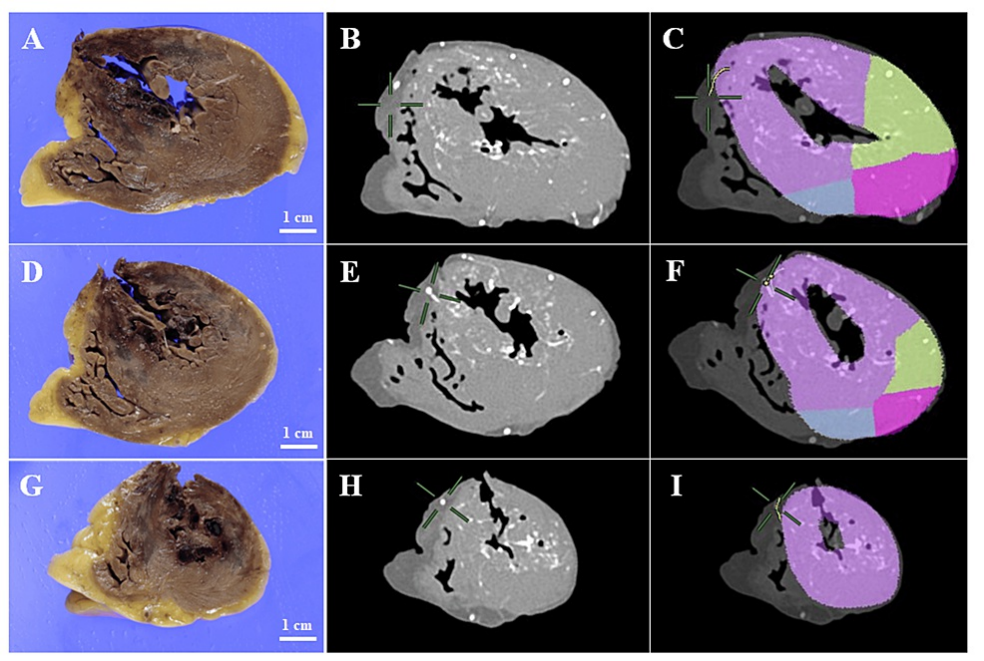
Comparison of macroscopic observation with postmortem computed tomography angiography (PMCTA) images. (A) Cross-section corresponding to the blue dashed line in Figure 2B. (B) Corresponding PMCTA image to panel A. (C) Computed tomography (CT) cross-section corresponding to panel B, with color coding of the perfusion territories: purple represents the ischemic region predicted based on the LAD stenosis, green indicates the perfusion territory of the LAD, pink indicates the perfusion territory of the LCX, and blue indicates the perfusion territory of the RCA. (D) Cross-section corresponding to the yellow dashed line in Figure 2B. (E) Corresponding PMCTA image to panel D. (F) CT cross-section corresponding to panel E, with color coding of the perfusion territories. (G) Cross-section corresponding to the green dashed line in Figure 2B. (H) Corresponding PMCTA image to panel G. (I) CT cross-section corresponding to panel H, with color coding of the perfusion territories. LAD: Left anterior descending artery

Coronary PMCTA was performed using a method according to those previously reported [4,10]. A 5% gelatin-barium emulsion radiopaque contrast medium was manually injected into the left coronary artery (LCA) and the right coronary artery (RCA) until the anterior and posterior descending arteries were visibly filled with a white emulsion. Then, the heart was fixed in a 10% phosphate-buffered formalin solution. Ten days after the autopsy, a CT scan of the heart was conducted using the following scan parameters: field of view, 18 cm; collimation, 0.5 mm; reconstruction interval, 0.5 mm; tube voltage, 120 kVp; 100 mA; rotation time, 0.75 s; and pitch factor, 0.75. The coronary PMCTA data were visualized and reconstructed using a Vincent workstation (Fujifilm, Tokyo, Japan). The maximum intensity projection (MIP) three-dimensional (3D) reconstructed images revealed significant filling defects in the left anterior descending artery (LAD) (Figures 1A, 1B). Correspondingly, two-dimensional (2D) CT axial images confirmed the presence of filling defects at the same site within the LAD artery (Figure 1C), indicating significant coronary artery stenosis. Macroscopic examination of the heart revealed a rupture of the left ventricular wall at the apex of the ventricular wall (Figures 2A-2C). Continuous cross-sections from the apex revealed an intramuscular hemorrhage and suspected necrosis, with wall thinning extending from the anterior wall to the septum of the left ventricle (Figures 3A, 3D, 3G). Reconstruction of CT cross sections corresponding to the macroscopic cross sections of the heart was done (Figures 3B, 3E, 3H) and the CT sections were color-coded (Figures 3C, 3F, 3I). Histopathological analysis showed features of coagulative necrosis, inflammatory cell infiltration, granulation tissue formation, and hemorrhage, corresponding to the macroscopic findings (Figure 2D). No significant changes were observed in the left ventricular lateral wall compared to the corresponding in the posterior wall, both macroscopically and microscopically. Narrowing of the LAD artery was also observed (Figure 2E). Histopathological examination confirmed plaque rupture and narrowing, with calcification (Figure 2F). These findings align with the occlusion observed in the 3D reconstructed images from the PMCTA data. Consequently, the likely cause of death in this case was cardiac tamponade secondary to myocardial infarction.

Comparison of ischemic regions using coronary PMCTA and macroscopic observation

**Figure 4:**
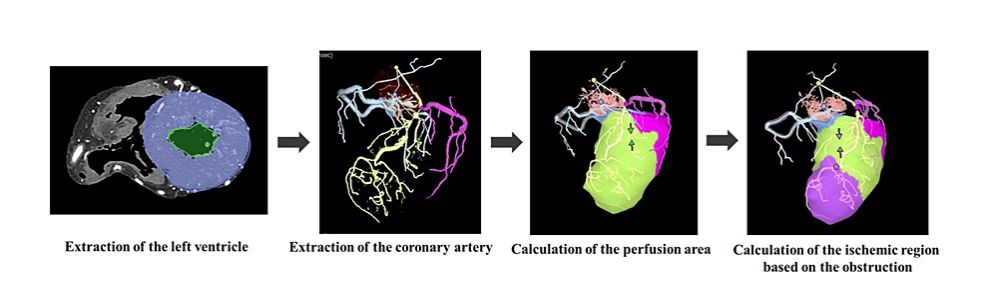
Workflow for predicting ischemic regions using coronary postmortem computed tomography angiography (PMCTA) analysis based on the Voronoi algorithm. The analysis begins by setting regions of interest in the myocardial regions of the left ventricle. The origins of the LCA and RCA were manually identified, followed by the semi-automatic extraction of the coronary arteries. The perfusion areas of each coronary artery were then calculated using the Voronoi algorithm. To predict the ischemic areas downstream of stenoses, the locations of stenotic segments observed in the anterior interventricular branch on maximum intensity projection (MIP) images were manually identified. Subsequently, the software automatically calculated the myocardial regions at risk of ischemia distal to the stenoses using the Voronoi algorithm. LCA: Left coronary artery; RCA: right coronary artery; LCX: left circumflex artery

The workflow for predicting ischemic regions using coronary PMCTA based on the Voronoi algorithm is depicted in Figure 4.

Coronary PMCTA images were analyzed using the CT workstation (Vincent, Fujifilm) with the "Cardiac Function and Coronary Artery Analysis Module." Initially, regions of interest were delineated within the myocardial regions of the left ventricle. The origins of the RCA and LCA were manually identified, followed by the semi-automatic extraction of the coronary arteries extending distally. The perfusion area for each coronary artery was computed using the Voronoi algorithm, which segmented the myocardium by assigning each voxel to the nearest coronary artery, thereby determining the perfusion territory of each artery.

To predict ischemic areas downstream of the stenoses, the locations of stenotic segments observed in the anterior interventricular branch on MIP images were manually identified. Subsequently, the software automatically calculated the myocardial regions at risk of ischemia distal to the stenoses based on the Voronoi algorithm.

The analysis revealed that the total myocardial volume of the left ventricle was 151.9 mL. The perfusion volume supplied by the LAD, left circumflex artery, and RCA was 92.9 mL (61.2% of the total), 34.2 mL (22.5%), and 24.9 mL (16.4%), respectively. The predicted ischemic volume distal to the stenosis was estimated to be 49.8 mL (32.8%).

To compare the ischemic areas observed macroscopically with the ischemic regions predicted from the coronary PMCTA images, CT cross sections corresponding to the macroscopic cross sections of the heart were reconstructed (Figures 3B, 3E, 3H). Additionally, these CT sections were color-coded to display the perfusion territories and predict the ischemic regions for each coronary artery (Figures 3C, 3F, 3I). These results demonstrated that the ischemic areas observed macroscopically were generally consistent with the predicted ischemic regions.

## Discussion

In this case, the macroscopic regions of myocardial infarction observed were generally consistent with the ischemic areas predicted from the coronary PMCTA images in a deceased patient who likely died from cardiac tamponade due to cardiac rupture secondary to myocardial infarction resulting from stenosis of the LAD. Cardiac rupture is a fatal mechanical complication of acute myocardial infarction (AMI), accounting for up to 15% of early deaths in patients with AMI [14,15]. Post-AMI cardiac tamponade, a complication of rupture, can involve the ventricular wall, septum, or papillary muscle [14]. Becker et al. identified three morphological types of cardiac rupture with free wall rupture. Type 1 rupture is characterized by an abrupt, slit-like myocardial tear corresponding to a recent infarct, usually occurring within 24 h. Type 2 ruptures show evidence of myocardial erosion, indicating a more gradual tear. Type 3 rupture involves marked myocardial thinning and perforation in the central portion of the aneurysm, typically occurring in late infarction lasting >7 days [16,17]. In this case, considering both the acute ischemic changes and the macroscopic thinning of the myocardial wall observed during autopsy, along with histopathological evidence of granulation tissue formation, it was predicted that the course of the condition was subacute, lasting a few weeks, corresponding to either type 2 or type 3 rupture.

Clinically, patients are likely to be referred for further examinations or revascularization procedures if the predicted ischemic area exceeds 10% of the left ventricular volume [11]. In the present case, the ischemic area predicted from the stenotic sites was 32.8% of the total left ventricular myocardium, which was presumed to be sufficiently lethal. However, further studies using coronary PMCTA are required to determine the exact percentage of lethal cases.

To our knowledge, this is the first case to suggest that the combination of coronary PMCTA and the Voronoi method enables accurate assessment of myocardial ischemic areas. This combination facilitates noninvasive visualization and quantification of perfusion and ischemic regions tailored to individual coronary artery anatomy. However, one limitation of the method for estimating myocardial ischemic areas using the Voronoi algorithm is that stenotic sites must be manually identified using CT images and histopathological examination. In this case, the stenotic sites were easily identified from 3D CT images. However, in PMCTA, distinguishing between artifacts caused by air and the actual stenosis can be challenging. Furthermore, as pointed out in multiple studies, one of the concerns with PMCTA is the potential to disrupt or dislodge thrombi that cause ischemia [8,10]. Further investigation is required to understand the impact of PMCTA on thrombus displacement. Moreover, the present study presents only one case; therefore, more studies with larger sample sizes are required. Future prospects for this method include its potential utility in accurately estimating perfusion territories and ischemic regions in cases involving anomalous coronary artery origin or course. Accumulating additional case studies would be beneficial for further validation and for enhancing broader clinical applicability.

## Conclusions

In conclusion, this case report is the first to demonstrate the effectiveness of combining PMCTA with the Voronoi algorithm to accurately assess myocardial ischemic areas. The ischemic regions predicted by PMCTA corresponded well with macroscopic observations during autopsy, highlighting the significant potential of this method for noninvasive visualization and quantification of ischemic myocardial regions. Further studies with larger sample sizes are needed to expand the applicability of this approach.
